# Cholinesterase Inhibitors: Drugs Looking for a Disease?

**DOI:** 10.1371/journal.pmed.0030140

**Published:** 2006-04-11

**Authors:** Marina Maggini, Nicola Vanacore, Roberto Raschetti

## Abstract

Maggini and colleagues examine the evidence on cholinesterase inhibitors for treating dementia. "What seemed a biologically plausible intervention," they say, "has not led to a proven, real improvement in patients' well-being."


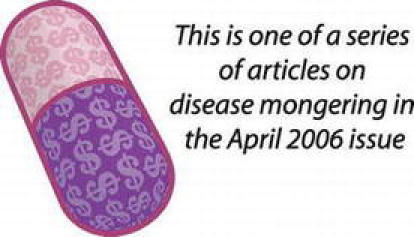


Randomized controlled trials (RCTs) are generally considered to be a robust form of evidence, free from bias, and the trial results are often used as a powerful tool to promote new drugs [ 1, 2]. However, because the inclusion criteria for many RCTs are often very restrictive (for example, trials generally exclude patients with serious concomitant illnesses) and because patients in trials tend to receive better care than those in standard-care settings, clinicians should be careful about generalizing RCT results to their own patients. Unfortunately, many drug treatments are widely used in clinical practice, sometimes beyond the approved indications, even when doubts remain about whether the results of RCTs of these drugs should be generalized. In this article, we discuss the use of cholinesterase inhibitors in patients with a variety of types of dementia and cognitive impairment, looking critically at the clinical trial evidence on these drugs.

If the results of these trials are not carefully evaluated, together with evaluating the methodological quality of the studies, this could lead to inappropriate prescribing of cholinesterase inhibitors. Drug companies have invested heavily in developing treatments for Alzheimer disease, and then were actively involved in expanding the market to other forms of dementia. In the last decade, donepezil, galantamine, and rivastigmine have been tested not only in patients with Alzheimer disease but also in patients with vascular dementia, dementia with Lewy bodies, dementia associated with Parkinson disease, and mild cognitive impairment (MCI). Even when the evidence on the efficacy of these drugs is lacking, or inconclusive, the results are often presented in such a way as to create a false perception of efficacy. For example, about 23 different scales or instruments (on average six per trial) were used, in the trials considered here, as primary or secondary outcome measures. Most of them were not validated for the disease for which the drugs were tested and are not currently used in clinical practice, undermining the translation of these research findings into clinical practice. Moreover, the treatment effect in the trials is usually expressed through the average change from baseline in test scores, without discussing the clinical importance of the usually small effect size observed.

## Alzheimer Disease: Waiting for New Treatments

The cholinesterase inhibitor donepezil was licensed in the US in December 1996, before the full results of clinical trials were available in medical journals [ 3]. The drug was launched with claims that it had produced “highly significant improvements in cognitive and clinical global assessments” in randomized trials lasting 30 weeks and had increased the proportion of “treatment successes” by 245% in patients with mild to moderate Alzheimer disease [ 3]. Donepezil, galantamine, and rivastigmine went on to be approved in many countries for the treatment of Alzheimer disease, even though it was clear that the efficacy, in the short term, was modest, symptomatic, and evident only in a subgroup of patients [ 4–8].

In a meta-analysis of randomized, double-blind placebo-controlled trials of cholinesterase inhibitors, Lanctôt and colleagues found that the pooled mean proportion of responders to drug treatment in excess of that for placebo treatment was only 10% (95% confidence interval, 4%–17%) [ 9]. In this study, response to therapy was defined (according to a definition first proposed by the US Food and Drug Administration) as an improvement of four or more points on the Alzheimer Disease Assessment Scale–cognitive portion (ADAS-cog) [ 10].

The most recent systematic review of RCTs, by Hanna Kaduszkiewicz and colleagues, analyzed the scientific evidence for the clinical use of cholinesterase inhibitors in Alzheimer disease, together with the methodological quality of the trials [ 11]. The authors concluded that the benefits are minimal, the methodological quality of the available trials is poor, and the scientific basis for recommendations of these drugs for Alzheimer disease is questionable [ 11].

A similar conclusion was reported in the preliminary draft of recommendations on the use of cholinesterase inhibitors that is being developed by the United Kingdom's National Institute for Health and Clinical Excellence (NICE), an independent organization responsible for providing national guidance on treating and preventing illness [ 12, 13]. In its preliminary draft appraisal document, the organization stated “that the RCT evidence on outcomes of importance to patients and carers, such as quality of life and time to institutionalisation, was limited and largely inconclusive.” Moreover, the NICE committee reported that the quality of the reviewed trials was mixed, and that “the assessment group suspected selection bias, measurement bias and attrition bias.” The preliminary recommendations of the appraisal committee were that “donepezil, rivastigmine and galantamine are not recommended for use in the treatment of mild to moderate Alzheimer's disease,” and that further research is required to identify subgroups of people for whom cholinesterase inhibitors may be effective. The committee recently updated its guidance, as shown in the Sidebar.

## Patients with Alzheimer Disease and Vascular Risk Factors or Patients with Vascular Dementia

The therapeutic potential of cholinesterase inhibitors has been explored in clinical trials of patients with Alzheimer disease with concurrent vascular risk factors, and also in patients with vascular dementia.

One 26-week placebo-controlled RCT evaluated the efficacy and safety of rivastigmine for patients with mild to moderately severe Alzheimer disease with or without concurrent vascular risk factors [ 14]. The authors concluded that the drug is effective in patients with or without vascular risk factors, and that those with vascular risk factors “experience greater clinical benefit (cognition, activities of daily living, and disease severity).” However, the withdrawal rate was higher for patients given the drug than for patients given placebo, and there was no intention-to-treat analysis.

The effect of galantamine was examined in a six-month RCT in a mixed population of patients diagnosed as having probable vascular dementia, Alzheimer disease with cerebrovascular disease, or an intermediate diagnosis [ 15]. Unfortunately, the study was not powered to detect treatment differences in the three subgroups; moreover, as in the study on rivastigmine [ 14], the primary statistical assessment of efficacy was not based on an intention-to-treat analysis, but only on an observed case analysis.

Two trials have been conducted to evaluate the efficacy and tolerability of donepezil in patients diagnosed with vascular dementia; these trials showed modest and inconsistent effects [ 16, 17]. The study design was similar to the design used in trials of cholinesterase inhibitors for Alzheimer disease: the vascular dementia trials used similar drug doses and similarly lasted only six months. As with trials of cholinesterase inhibitors for Alzheimer disease, a six-month trial period is unjustified for a pathology that develops over decades. Moreover, the assessment scales used in the vascular dementia trials are intended for assessing Alzheimer disease, and are not validated for the evaluation of vascular dementia. The investigators did not find improvement for all primary and secondary efficacy parameters, and a reverse dose effect was shown: that is, improvement in global function was observed in a greater proportion of patients treated with donepezil than those treated with placebo in the 5-mg group but not in the 10-mg group [ 16].

The study population was, as reported by the authors, not typical of all patients with vascular dementia (in fact, only patients who were stable with respect to comorbid conditions, hypertension, diabetes, and heart disease were included in these clinical trials) [ 16]. Even in this highly selected population, an excess of stroke (fatal and nonfatal) was observed among treated patients. The potential implications for clinical practice still remain to be clarified. Nevertheless, the drug was presented in the trial reports as a safe and effective means of treating vascular dementia. After a pooled analysis of the two trials, the authors wrote that “the results … are somewhat confusing,” and “further data on donepezil's impact on executive functioning would be certainly desirable” [ 18, 19].

At the time of writing this article, the data from these vascular dementia trials have not been considered sufficient evidence to license donepezil for treating vascular dementia. However, the positive messages contained in the published RCTs may promote the off-label use of the drug.

## Dementia Associated with Parkinson Disease and Dementia with Lewy Bodies

A Cochrane systematic review identified only one RCT (involving 120 patients) of the efficacy of rivastigmine in patients with probable dementia with Lewy bodies [ 20, 21]. The Cochrane reviewers concluded that the trial “showed no statistically significant difference between the two groups at 20 weeks. A possible beneficial effect on neuropsychiatric features was found only in analysis of observed cases, and may therefore be due to bias.” Hence the evidence of any benefit is currently weak [ 21].

Two clinical trials have investigated the effect of cholinesterase inhibitors in patients with dementia associated with Parkinson disease. The first one [ 22], which found a trend (not statistically significant) toward better scores on the ADAS-cog is not further discussed here because of its small size (only 22 patients were randomized to receive donepezil or placebo).

The second trial, by Emre et al., investigated the effect of rivastigmine in 541 highly selected patients recruited from an unspecified number of centers from 12 countries [ 23]. Patients included in the trial had received a diagnosis of dementia 6.6 ± 5.2 years (treated arm) and 7.3 ± 5.2 years (placebo arm) after the diagnosis of Parkinson disease. It would be difficult to find such a population in a clinical setting for a number of reasons. Beyond the diagnostic challenge of differentiating dementia associated with Parkinson disease from dementia of the Lewy body type, there is also evidence that the risk of dementia in Parkinson disease is associated with age and severity of extrapyramidal signs, and the mean time from onset of Parkinson disease to dementia is estimated to be 10.5 years [ 24–26]. But the exact clinical implications of this RCT are still not clear.

The outcome measures used in Emre and colleagues' trial were the ADAS-cog and the Alzheimer Disease Cooperative Study–Clinician's Global Impression of Change scale.

In their trial, the authors considered a mean improvement of 2.25 points in the ADAS-cog score as clinically meaningful, even though this scale has never been used to monitor the progression rate of dementia in Parkinson disease. Among adverse events, Parkinsonian symptoms were reported more frequently in the rivastigmine group than in the placebo group. The authors concluded that rivastigmine was associated with moderate but significant improvements in all symptoms of dementia associated with Parkinson disease, but also with high rates of adverse events, and that the findings may have implications for clinical practice. But the exact clinical implications of this RCT are still not clear.

Clinicians should be careful about generalizing RCT results to their own patients.

## Mild Cognitive Impairment: A New Clinical Entity or a New Market Frontier?

Whether MCI can be considered a clinical entity is still a matter of debate (for example, Gauthier and Touchon have argued that “there is epidemiological evidence that many subjects labeled as having MCI do not worsen over time and may revert to normal cognitive abilities” [ 27]). Nevertheless, specific drug treatment for MCI has been proposed.

Two RCTs have been conducted to investigate whether donepezil delays the onset of dementia in people with MCI. These studies failed to demonstrate any efficacy, while showing a worse safety profile among patients receiving active drug compared with the placebo group. In the first published trial [ 28], significant treatment effects were not seen in the primary efficacy measures, while more patients treated with donepezil experienced adverse events compared with patients treated with placebo (88% versus 73%). Despite this negative result, a new trial was conducted by Petersen et al., comparing donepezil, vitamin E, and placebo [ 29]. This study did not show a significant difference among the three groups in the rate of progression from MCI to Alzheimer disease over a three-year period. Nevertheless, the authors stress some limited effects on secondary measures: a reduced likelihood of progression to Alzheimer disease only during the first 12 months of treatment, and a benefit of donepezil among carriers of one or more apolipoprotein E ɛ4 throughout the three-year follow-up. This latter claim, in particular, was not supported by the data as the study was not statistically powered to evaluate the effect of the treatment in separate groups of apolipoprotein E ɛ4 carriers.

Harms-related data were inadequate: the flow of participants through the study phases was not described; the reasons and timing for discontinuation per treatment arm were not reported; only adverse events observed in at least 5% of patients were reported; and the causes of the 23 deaths observed (17 in the double-blind phase and six in the subsequent open-label phase) were not specified. In the double-blind phase, a higher number of deaths was observed in the donepezil arm ( *n* = 7) compared with the vitamin E arm ( *n* = 5) and the placebo arm ( *n* = 5). For the six deaths that occurred during the open-label phase, the original arm (active drug or placebo in the previous double-blind phase) was not reported. (The distribution of these six deaths across the three arms of the trial in the open phase was subsequently reported by Jelic et al. [ 30]—there were three deaths in the donepezil group, one in the vitamin E group, and two in the placebo arm; thus, the total number of deaths per arm in the whole trial was ten in the donepezil group [three from cardiac arrest], six in the vitamin E group, and seven in the placebo group.) Although Petersen et al. conceded that the results “do not provide support for a clear recommendation for the use of donepezil in persons with mild cognitive impairment,” they did suggest that their findings “could prompt a discussion between the clinician and the patient about this possibility” [ 29].

Two trials, each lasting two years and not yet published, evaluated the effect of galantamine on a total of 2,048 patients with MCI randomized to receive galantamine or placebo [ 31, 32]. Overall, the studies did not show that the drug could improve cognition or delay the conversion to dementia. Increased mortality (mostly due to myocardial infarction and stroke) was observed among patients treated with galantamine compared with patients given placebo. On the basis of these results, the US Food and Drug Administration issued a safety warning concerning galantamine [ 33].

In these trials, the treatment duration (two years) was longer than that of most previous RCTs on Alzheimer disease (typically only six months). The short trials on Alzheimer disease had shown no increased mortality associated with cholinesterase inhibitors compared with placebo. In clinical practice, though, these drugs would likely be prescribed for several years, and the galantamine trials [ 31, 32] have shown that such prolonged use may be associated with increased mortality. A recent review on clinical trials in MCI concluded that none of the reviewed studies met their primary objectives; that is, none of the trials showed a benefit of cholinesterase inhibitors in delaying the conversion to dementia or in slowing symptom progression [ 30].

## Conclusion

At present, donepezil, galantamine, and rivastigmine are licensed only for the treatment of mild to moderate Alzheimer disease. The treatment effect is modest, and there is evidence of wide variability in the outcomes reported: “some patients will have improved, others stayed the same, while others will have deteriorated. This variance should be comparative in both the treatment and the placebo groups but care should be taken over the interpretation of the mean scores” [ 34].

However, a minority of people with Alzheimer disease may benefit from the cholinesterase inhibitors, and further research is needed to identify these subgroups of people, considering, in particular, long-term and worthwhile improvements such as delay in institutionalization. A cohort study of the effectiveness of cholinesterase inhibitors in Alzheimer disease has been conducted in Italy on 5,462 patients [ 35]. This study showed that the patients most likely to respond to treatment are those without concomitant diseases and those who had demonstrated an early response at three months. Response to treatment did not vary among groups with different Mini Mental State Examination (MMSE) scores at baseline. Based on these results, we suggest that physicians should accurately reevaluate their patients after three months of therapy, and should communicate realistic information to patients and their families about the very modest benefits of these drugs.

Since 1996, when the first cholinesterase inhibitor was licensed in the US for the symptomatic treatment of Alzheimer disease, each new published trial on the effect of cholinesterase inhibitors on the various different forms of dementia has raised new questions about the benefit–risk profile of these drugs. Reduced cholinergic neurotransmission was the rationale for the use of cholinesterase inhibitors in patients with dementia. Nevertheless, what seemed a biologically plausible intervention has not led to a proven, real improvement in patients' well-being.

**Search Strategy**

For this article, we searched the MEDLINE database from 1996 to 2005 using the terms donepezil, galantamine, and rivastigmine to find randomized controlled clinical trials, systematic reviews, and meta-analyses. Our article is not itself a systematic review, but we discuss all the major RCTs, systematic reviews, and meta-analyses of these drugs as treatments for Alzheimer disease, and we discuss the major RCTs of these drugs for other forms of dementia.

**NICE Recommendations on Cholinesterase Inhibitors**

Revised draft guidance on the use of drugs to treat Alzheimer disease has recently been published (23 January 2006) on the NICE Web site ( http://www.nice.org.uk/page.aspx?o=288826).

The preceding draft guidance from NICE ( http://www.nice.org.uk/page.aspx?o=245908), published 1 March 2005, concluded that there was not enough evidence to support the use of these drugs for all patients. However, responses received from stakeholders during consultation on this first draft suggested that the drugs may be more effective for certain groups of people. NICE, therefore, asked the pharmaceutical companies involved in the appraisal to look for evidence to support this, from the data in their clinical trials.

In conclusion, “the Committee considered not just the initial evidence and submissions, but also the comments raised in consultation on the first Appraisal Consultation Document (notably the improved infrastructure around dementia care) and the evidence that was submitted during consultation and the additional analyses undertaken. The Committee concluded that taking all these factors into account, the resulting estimates of cost effectiveness could be considered sufficiently acceptable to allow the prescribing of AChE inhibitors,” donepezil, galantamine, and rivastigmine, for people with Alzheimer's disease of moderate severity only (that is, those with an MMSE score between ten and 20).

As in the earlier draft, the committee “noted, however, that the evidence available on the long-term effectiveness of the AChE inhibitors on outcomes of importance to people with Alzheimer's disease and their carers, such as quality of life and delayed time to nursing home placement, was limited and largely inconclusive.”

As for memantine, it continued to be “not recommended as a treatment option for people with Alzheimer's disease except as part of properly constructed clinical studies.”

## Abbreviations

ADAS-cog: Alzheimer Disease Assessment Scale–cognitive portion
MCI: mild cognitive impairment
MMSE: Mini Mental State Examination
NICE: National Institute for Health and Clinical Excellence
RCT: randomized controlled trial
